# Clinical examination, MRI and arthroscopy in meniscal and ligamentous knee Injuries – a prospective study

**DOI:** 10.1186/1749-799X-3-19

**Published:** 2008-05-19

**Authors:** TR Madhusudhan, TM Kumar, SS Bastawrous, A Sinha

**Affiliations:** 1 Registrar, Department of Orthopaedics, Glan Clwyd Hospital, Bodelwyddan, North Wales, LL18 5UJ, UK; 2 Associate specialist, Department of Orthopaedics, Glan Clwyd Hospital, Bodelwyddan, North Wales, LL18 5UJ, UK; 3 Consultant orthopaedic surgeons, Department of Orthopaedics, Glan Clwyd Hospital, Bodelwyddan, North Wales, LL18 5UJ, UK

## Abstract

Data from 565 knee arthroscopies performed by two experienced knee surgeons between 2002 and 2005 for degenerative joint disorders, ligament injuries, loose body removals, lateral release of the patellar retinaculum, plica division, and adhesiolysis was prospectively collected. A subset of 109 patients from the above group who sequentially had clinical examination, MRI and arthroscopy for suspected meniscal and ligament injuries were considered for the present study and the data was reviewed. Patients with previous menisectomies, knee ligament repairs or reconstructions and knee arthroscopies were excluded from the study. Patients were categorised into three groups on objective clinical assessment: Those who were positive for either meniscal or cruciate ligament injury [group 1]; both meniscal and cruciate ligament injury [group 2] and those with highly suggestive symptoms and with negative clinical signs [group 3]. MRI was requested for confirmation of diagnosis and for additional information in all these patients. Two experienced radiologists reported MRI films. Clinical and MRI findings were compared with Arthroscopy as the gold standard. A thorough clinical examination performed by a skilled examiner more accurately correlated at Arthroscopy. MRI added no information in group 1 patients, valuable information in group 2 and was equivocal in group 3 patients. A negative MRI did not prevent an arthroscopy. In this study, specificity, positive and negative predictive values were more favourable for clinical examination though MRI was more sensitive for meniscal injuries. The use of MRI as a supplemental tool in the management of meniscal and ligament injuries should be highly individualised by an experienced surgeon.

## Introduction

Clinical tests used in the diagnosis of meniscal and cruciate ligament damage have limitations and it may not be possible to elicit objective signs repeatedly, more so in a busy orthopaedic clinic and being painful in an acute or sub acute presentation. An accurate clinical diagnosis requires experience although difficult to quantify. Magnetic resonance imaging [MRI] has revolutionised the diagnosis and management of intra-articular pathology and ligamentous injuries. Being non invasive and a highly sensitive tool of investigation, early and subtle changes in the soft tissues often are picked up by MRI. Arthroscopy being highly sensitive and specific procedure is both diagnostic and therapeutic, but is invasive.

The aim of this study was to correlate the different modalities of diagnosis with arthroscopy as the gold standard and whether a negative MRI could justifiably deny an arthroscopy.

## Patients and methods

Data from 565 consecutive knee arthroscopies performed by two experienced knee surgeons between 2002 and 2005 for degenerative joint disorders, ligament injuries, loose body removals, lateral release of the patellar retinaculum, plica division, and adhesiolysis were prospectively collected. From the above data, a subset of 109 patients who sequentially had clinical examination, MRI and arthroscopy for suspected meniscal and ligament injuries were considered for the present study and the data was reviewed. Patients with previous menisectomies, knee ligament repairs or reconstructions and knee arthroscopies were excluded from the study.

Clinical data including patient demographics, wait period between MRI and arthroscopy, suggestive symptoms including effusion, presence of a "pop", locking, mechanism of injury, clinical diagnosis, and operative details were documented and analysed. All patients were examined by two experienced orthopaedic consultants. Clinical tests included Mcmurrays' for meniscal damage, Draw tests for cruciate damage, and valgus and varus stress tests for collateral ligament integrity. A clinical diagnosis was made and an MRI of the affected knee was requested in all 109 patients. MRI was requested for confirmation of clinical diagnosis and for obtaining additional information.

MRI was performed with a dedicated magnetic extremity coil of 1 tesla strength. Each film provided 19 slices of T1 and T2 images of 4 mm thickness and 160 mm field of view. The radiologists were provided patient identifying data, and the provisional clinical diagnosis. Two experienced radiology consultants reported on all the MRI scans. MRI films and reports were retrieved from the Synapse software system. Arthroscopies were performed under Spinal or general anaesthesia as appropriate. Operative findings were documented in the operation theatre, which included the anatomical structure involved with the presence or absence of tear, its location, status of the articular cartilage and additional details when available. The composite data was tabulated on Microsoft excel spreadsheet and studied for correlation.

There were three identified groups: Those who were clinically positive for meniscal or cruciate ligament injury [group 1], combined meniscal and cruciate ligament injury [group 2], and patients with highly suggestive symptoms but with negative clinical tests [group 3].

Full agreement was when the modalities correlated accurately. Any disparity between clinical examination and MRI at arthroscopy was considered no agreement. Partial agreement was when there was partial correlation between the modalities. True positives and True negatives were calculated from the clinical diagnoses and arthroscopic correlations and MRI and arthroscopic correlations for meniscal and anterior cruciate ligaments (ACL). A true-positive result had an abnormal finding (meniscus, ACL) reported by MRI and confirmed at arthroscopy surgery. A true negative-result had no abnormalities noted clinically or by MRI or at Arthroscopy. A false positive was considered if the clinical examination or MRI reported an abnormality but was not confirmed at arthroscopic operation. A false-negative result had a negative clinical examination or MRI report and a positive finding at operation.

Sensitivity (True-positives × 100/[True-positives + false-negatives]), Specificity (True-negatives × 100/[True-negatives + false-positives]), Positive predictive value (True positives × 100/[True-positive + false-positives]), Negative predictive value (True-negatives × 100/[True-negatives + False-negatives]) were calculated from the data. Correlation of clinical examination and MRI with Arthroscopy from the pooled data of 109 patients was expressed as a percentage.

## Results

There were 68 males and 41 female patients in the age group of 18–70 years with a mean age of 52 yrs. Patients in groups 1 and 2 were in the age group of 18 and 50 years and group 3 consisted of 62 patients in the age group of 41–70 years. 82 patients in the study had treatment in the form of a knee support device or physiotherapy prescribed by their general practitioner before their first visit to the orthopaedic consultation. The patients had received symptomatic treatment for 16–43 days, [Average 26 days]. 3 patients were examined directly by the orthopaedic team following an acute episode and the rest were seen by the emergency medicine department at the time of injury to be followed by Orthopaedic consultation.

The waiting time for the MRI from the point of definite clinical diagnosis was 3–7 weeks [average 4.1 weeks] and the waiting time for arthroscopy following the MRI was a further 5–8 weeks [average 5.8 weeks]. There were no episodes of fresh or repeat injuries during either of these wait periods.

In Group 1 there were 33 patients. There were 21 patients with meniscal injuries and 12 patients with cruciate ligament injuries. 12 patients were positive for medial meniscus and 9 patients for lateral meniscus injuries clinically. MRI and Arthroscopy fully confirmed the meniscal tear in 20 patients. In the remaining one patient, arthroscopy did not confirm the presence of a meniscal tear. 12 patients were positive for anterior cruciate ligament injury clinically. MRI confirmed tear in 7 patients fully and partially agreed in 4 patients. In the remaining one patient, there was no correlation. Arthroscopy confirmed ACL injury in all the 12 patients and a partial tear of the posterior cruciate ligament in one knee.

In group 2, there were 14 patients with combined ligament injuries.6 patients were positive for medial meniscus and anterior cruciate ligament injuries, 3 patients with medial and lateral meniscus, anterior cruciate and lateral meniscus in 3 and anterior cruciate ligament, medial and lateral menisci 2 patients. MRI fully agreed in 6 patients with medial meniscus and anterior cruciate ligament injuries and in 2 patients with both menisci injuries. In the rest 6 patients there was no correlation but MRI suggested additional information in 5 patients. Arthroscopy fully agreed with clinical examination and MRI in 6 patients with medial meniscus and anterior cruciate ligament injuries. There was no anterior cruciate ligament injury in 1 patient and partially agreed with MRI in 5 patients.

In group 3, there were 62 knees with highly suggestive symptoms of an intra articular pathology but were negative on clinical examination. All patients had either one or more symptoms, which included persistent pain, locking, and recurrent swelling of the knees and instability. Three subgroups were further identified.

a) 24 knees were reported to have posterior horn meniscal tears (13 for medial meniscus and 11 for lateral meniscus) 14 of which confirmed at arthroscopy.

b) 25 patients were normal on MRI but had lateral meniscus tears at Arthroscopy.

c) 9 patients had cartilage damage and 4 had synovial plicae.

2 patients with cartilage damage were symptomatic on follow up clinics and those who had the plicae removed were relieved of the symptoms. The results and the correlation between the three modalities in all the groups have been summarised in tables 1 to 4. The extent of correlation, sensitivity, specificity, positive and negative predictive values between the modalities from the pooled data of 109 patients are as per tables 4, 5  and 6.

**Table 1:** Clinical examination Vs Arthroscopy (Groups 1 and 3)

	**Full Agreement**				**No agreement**				**Comments**
**Group 1 n= 33**	**MM**	**LM**	**ACL**	**PCL**	**MM**	**LM**	**ACL**	**PCL**	Additional PCL damage in 1 patient on arthroscopy
	12	9	12	0	0	0	0	0	
**Group 3 n= 62**	10	3	-	-	13	36	-	-	

n = Number of patients, MM = medial meniscus, LM = lateral meniscus, ACL = Anterior cruciate ligament, PCL = Posterior cruciate ligament

**Table 2:** MRI Vs Arthroscopy (Groups 1 and 3)

	**Full Agreement**				**Partial agreement**				**No agreement**				**Additional information**	
**Group 1 n= 33**	MM	LM	ACL	PCL	MM	LM	ACL	PCL	MM	LM	ACL	PCL	Cartilage	Plicae
	12	9	7	0	1	0	0	4	0	0	0	0	0	0
**Group 3 n= 62**	10	4	0	0	0	0	0	0	7	28	0	0	9	4

n = Number of patients, MM = medial meniscus, LM = lateral meniscus, ACL = Anterior cruciate ligament, PCL = Posterior cruciate ligament

**Table 3:** Clinical Examination Vs Arthroscopy (Group 2)

	**Full Agreement**			**Partial agreement**			**Comments**
**Group 2 n= 14**	**MM + ACL**	**MM + LM**	**ACL +LM**	**MM + ACL**	**MM + LM**	**ACL+LM**	cartilage damage in 5 patients
	7	1	2	2	1	1	

n = Number of patients, MM = medial meniscus, LM = lateral meniscus, ACL = Anterior cruciate ligament, PCL = Posterior cruciate ligament

**Table 4:** MRI Vs Arthroscopy (Group 2)

	**Full Agreement**			**Partial agreement**		
**Group 2 n = 14**	**MM + ACL**	**MM +LM**	**ACL+LM**	**MM + ACL**	**MM +LM**	**ACL+LM**
	7	2	3	1		1

Comparison of agreement between clinical examination, MRI and Arthroscopy findings among the 109 patients						

	**Full agreement**	**Partial agreement**	**No agreement**			

**Clinical vs. Arthroscopy**	43(39.44%)	14(12.84%)	52(47.70%)			
**Clinical vs. MRI**	66(60.55%)	19(17.43%)	24(22.01%)			
**MRI vs. Arthroscopy**	54(49.54%)	20(18.34%)	35(32.11%)			

n = Number of patients, MM = medial meniscus, LM = lateral meniscus, ACL = Anterior cruciate ligament, PCL = Posterior cruciate ligament

**Table 5:** Clinical examination correlation (percentage) with Arthroscopic findings by type of Injury among the 109 patients

**Test**	**Meniscus**	**ACL**
**Sensitivity**	38.75	100
**Specificity**	93.10	97.93
**Positive predictive value**	93.93	85.71
**Negative predictive value**	35.32	97.93

**Table 6:** MRI correlation (percentage) with Arthroscopic findings by type of Injury among the 109 patients

**Test**	**Meniscus**	**ACL**
**Sensitivity**	59	54
**Specificity**	50	91.83
**Positive predictive value**	75.8	42.85
**Negative predictive value**	31.91	94.73

ACL = Anterior cruciate ligament

## Discussion

In the United Kingdom, patients with a suspected ligament or meniscal damage are often seen in the accident and emergency department or peripheral clinic or the general practitioner in the first instance. A symptomatic treatment in the form of a knee support device or physiotherapy is offered until seen by a specialist and a definitive treatment is planned. This approach may reduce the pain and make subsequent clinical examination easier and more conclusive. On rare occasions the patient is seen directly by the concerned specialist.

The demographics of the population focused in our study were comparable and more than 50% were in the 4^th ^and 5^th ^decade. With increasing life expectancy and activity levels, we believe this age group will be a major subset of population seen in orthopaedic clinics in the UK.

A good history with particular reference to the nature of injury and a well-performed clinical examination will in most situations indicate the underlying problem. This is improved by experience, and arthroscopy may be justified on clinical grounds alone [1]. Though the accuracy of clinical diagnosis of meniscal and ligament injuries has been varied in the literature [2,3], a thorough clinical examination carried out by an experienced examiner in most situations will indicate the nature of the intra-articular injury. Clinical examination is as accurate as MRI and MRI should be reserved for confusing and special cases [4].

The decision to use an expensive investigative tool like MRI should be based on the criteria that the test will confirm or expand the diagnosis or change the diagnosis in such a way that this is going to alter the proposed treatment. It should supplement to formulate a therapeutic decision as well [5]. This entirely rests on the treating physician. In unclear situations, the clinician requests an MRI for additional information to aid plan the operation and to predict the prognosis. This is compounded by high patient expectations, high degree of awareness amongst the public and availability of MRI in most district general hospitals in the UK. A wait period for an MRI and a definitive arthroscopy thereafter is inevitable considering the load in the National Health Service (NHS).

In knees with multiple ligament injuries, the diagnostic specificity of MRI for ligament tears decreases, as does the sensitivity for medial meniscus tears [6]. MRI added valuable information in 4 clinically confirmed patients which helped the surgeon for better planning. MRI is useful but should be reserved for situations in which an experienced clinician requires further information before arriving at a diagnosis [7]. Our observations agree with the above findings.

Though MRI has been recommended as a clarifying diagnostic tool [8], as in other studies we found MRI added little information to an already established clinical diagnosis [9]. Interestingly in our study, patients in whom all the modalities fully agreed consisted of younger patients. Those with highly suggestive symptoms but with negative clinical tests had arthritic changes on plain radiographs, which were confirmed at arthroscopy. An accurate examination may be difficult even for an experienced examiner in this situation and it may be that an arthritic knee may not allow a complete examination. A conclusive diagnosis was therefore not possible. This may account for the low sensitivity of clinical tests in our study. In these situations, the value of MRI is heightened and invariably is requested for confirming the diagnosis.

In the middle aged and elderly patients a lower threshold of suspicion is warranted for meniscal tears as they follow minor trauma [10] and MR signal alterations are significantly higher in older population [11]. MRI accuracy depends to a large extent on the structure studied, technical factors including imaging parameters, coil strength, surface coil use and planes of image [5]. Partial tears of ACL may be identified as an altered signal alone and imaging may not be accurate due to the overlying synovial reaction [5]. Further, the sensitivity of MRI for medial and lateral menisci being different there would be many lateral meniscal tears being missed and medial meniscal tears being over diagnosed [3]. A high reliability on the MRI for a diagnosis and additional information will in these situations be a futile attempt [9]. We agree with the above findings. A sound clinical judgment and experience is therefore required in the presence of a normal MRI. However the decision to do an arthroscopy was already made in these patients considering the clinical picture and MRI scans in these patients would have misled the surgeon into not doing an arthroscopy.

Cartilage lesions have not been addressed in the present study. Earlier studies suggested that MRI has a doubtful value in cartilage lesions [8]. Even though un-enhanced MRI using a 1.5-Tesla magnet with conventional sequences (proton density-weighted, T1-weighted, and T2-weighted) is most accurate at revealing deeper lesions and defects at the patellae, a considerable number of lesions will remain undetected until arthroscopy [12]. MRI scans with 3-Tesla field strength however improves the visualisation of hyaline cartilage with comparatively good diagnostic values but the positive predictive values remains low for all grades of lesions. [13]. In our study, there were no traumatic cartilage lesions and most of the cartilage tears were degenerate and superficial, though we did not attempt to classify the tears as it was beyond the scope of the present study. MRI scans with 1 tesla field strength as in our study failed to highlight these tears in most of our patients accounting for a low sensitivity and specificity, which would perhaps been picked up by a higher field strength MRI scan. High quality MRI films may therefore still be useful in delineating the anatomical location and the geometry of the tear, as treatment options differ. This would thus help the surgeon in better planning but may not completely avoid an arthroscopy procedure. We presume that the plicae were symptomatic in a few patients as the symptoms resolved following removal.

Reports from radiology literature have highlighted the importance of quality reporting by experienced musculoskeletal radiologists [14-16]. To be of value, MRI of the knees should follow a specific protocol and should be performed and reported by experienced musculoskeletal radiologists [5]. For practical reasons, it may not be possible to have a specialised musculoskeletal radiologist in all district general hospitals in the UK. With these subjective and inherent factors influencing the outcome of MRI report, it would seem unrealistic to base the decision to deny an arthroscopy on a negative MRI alone. As in other studies a negative MRI did not prevent us from doing an arthroscopy [5].

We recognise the limitations of this study in terms of the small numbers but believe that the groups studied are representative of the population normally attending the orthopaedic clinics.

## Conclusion

An accurately performed clinical examination by an experienced examiner with positive signs alone will be justified for arthroscopy. A normal MRI will not be a sufficient evidence to deny an arthroscopy particularly in individuals with arthritic knees. The use of MRI as a supplemental tool for clinical decision-making should be highly individualised.

## Authors' contributions

TRM is the principal author and was responsible for study design, data collection, analysis and interpretation, and drafting the manuscript. TMK and SSB were involved in proofreading the manuscript. ASI participated in the study design and co ordination and proof-read the manuscript. All authors read and approved the final manuscript.
